# New psychoactive substance α-PVP in a traffic accident case

**DOI:** 10.1007/s11419-016-0309-x

**Published:** 2016-04-02

**Authors:** Sebastian Rojek, Karol Kula, Martyna Maciów-Głąb, Małgorzata Kłys

**Affiliations:** Department of Forensic Medicine, Jagiellonian University Medical College, Grzegórzecka 16 Str, 31-531 Kraków, Poland

**Keywords:** Traffic accident, New psychoactive substances (NPSs), α-pyrrolidinovalerophenone (α-PVP), Psychological symptoms, DRUID

## Abstract

The problems of new psychoactive substances (NPSs), especially related to drivers, constitute an open research area. In this case report, we present a traffic accident case, in which two passengers of five individuals died instantly, while the other three persons survived the accident with minor injuries only. From the blood samples of the driver and the passengers, α-pyrrolidinovalerophenone (α-PVP), an NPS belonging to the category of cathinone derivatives, was disclosed. Therefore, we established a detailed procedure for analysis of α-PVP in blood samples by liquid chromatography–tandem mass spectrometry. After careful validation tests of this method, α-PVP concentration in blood samples from the surviving driver and passengers, and from the two deceased, were measured. The concentrations varied from 20 to 650 ng/mL. Access to detailed information originating from the court files and from explanations provided by the driver and eye witnesses revealed extremely valuable illustrative details addressing the symptoms and pharmacological effects of α-PVP on the human organism, thus contributing to enriching the body of knowledge of α-PVP abuse.

## Introduction

The issues associated with driving under the influence of alcohol and narcotic substances are subject to continuous interest not only for legal practitioners, but also for the police, prosecution, and medico-legal teams. Among initiatives implemented in this field, there is the European Union DRUID program carried out by 19 EU countries in the years 2006–2011, aiming at evaluating the phenomena. The final report claimed that although the gravest danger in traffic in EU member countries was posed by alcohol consumption, especially when combined with medications, considerable problems also arose by other psychoactive substances, such as amphetamines and cannabis that are the most popular [1].

Practice shows that preliminary testing of a driver by police road patrol to detect the presence of alcohol and classic narcotic substances often yields a negative result; the driver may be under the influence of other psychoactive agent(s), the presence of which is not detected by most of testers. This may cause the driver to develop a sense of impunity and thus leads to increased chance of traffic accidents. Inasmuch as the issue of alcohol and classic narcotic substances in relation to traffic has been legally regulated in keeping with legislative directives of particular European countries, the problem of new psychoactive substances (NPSs) as related to drivers constitutes an open research area. Although research papers addressing this subject have been published [2–6], the problem continues to be poorly investigated.

In this case report, we present a traffic accident case in which two passengers were killed, and the driver and two passengers survived; from the blood samples of all individuals, variable concentrations of α-pyrrolidinovalerophenone (α-PVP), an NPS belonging to the category of cathinone derivatives, were detected. Access to detailed information originating from the court files and derived from explanations provided by the driver and the eye witnesses revealed extremely valuable illustrative material addressing the effect of α-PVP on the human organism, thus contributing to enriching the body of knowledge of the NPS.

## Case history

According to the court files, in a small town in Poland, a group of young people was driving home from a nearby discotheque in early morning hours. Approximately at 6 a.m. the driver, exercising no special caution, drove onto the curb of a safety island, lost control of the car, and drove into a ditch, where the car overturned and hit a tree. In consequence of the accident, two passengers, a 17-year-old female and a 28-year-old male, were killed instantly; one of the remaining passengers, a 19-year-old female, suffered a minor spine injury, while another passenger, a 36-year-old male, and the 21-year-old driver succumbed to minor injuries. Immediately upon arrival of the police, the surviving individuals involved in the accident, the driver and the passenger sitting in front were tested for state of intoxication by means of breath analyzers. The wounded female passenger was tested upon arrival at a hospital ~1 h later, while the deceased individuals were tested by means of postmortem blood analysis. After the accident, blood samples were collected and analyzed for psychoactive substances, with the exception of the injured 19-year-old female, whose blood was tested for ethyl alcohol upon admission. As follows from the court files, all people involved in the accident except the driver were under the influence of alcohol, which was later confirmed by tests.

The sensations felt by the driver following his taking the narcotic substance were described on his allegation. The psychoactive substance was insufflated through the nose by him and his friends. They had a form of crystals called FETA, KATAR, or KRYSTYNA. The persons attending the party had taken the first fix of the psychoactive substance ~6 h prior to the accident. The driver informed that the fix of the substance in an amount of ~0.4 g “was good” for about 6 h. One person had prepared the so-called toot, which was then divided and insufflated through the nose. The doses were not calculated, but rather applied using a “hit-and-miss” method. Having insufflated the first fix of the substance, the driver felt agitated, started to sweat profusely, and experienced palpitations. He exploded with energy in spite of the fact that he was tired, having previously performed physical labor in the forest. He had not taken any alcohol. Together with his friends, he took another fix 4 h later, i.e., ~2 h prior to the accident. Afterwards, he was agitated, no longer sleepy, and did not experience palpitations or sweating; he was only thirsty, but generally felt well and full of inner energy. Immediately before the accident, however, he recounted: “…suddenly I saw spots before my eyes…for a moment I felt as if I had lost consciousness, when I snapped out, there was a safety island in front of me…then I jerked the steering wheel to the right and to the left…. I put the car in neutral…I pressed the gas and clutch…I did not use brakes…I could not control the car…”. Then everything went very fast. The car overturned. The passengers sitting in the backseat occupying side seats, without their seat-belts fastened, died instantly, the remaining individuals sustained minor injuries.

It was obvious that the driver and the fault that he committed would be burdened by the highest degree of guilt for causing the accident; nevertheless, according to the information available, the other individuals present in the car and involved in the accident might contribute to its happening indirectly. At the discotheque, through various forms of pressure, the driver was forced by the other participants to drive, because he had not drunk alcohol. However, he did not have a driving license. During the drive, the passengers were agitated, and they shouted to pressure the driver into going faster.

Addressing a question to experts whether the presence of α-PVP at the level determined could have affected the psychomotor abilities of the driver leading to the miserable accident, the public prosecutor’s office expected a justified and positive answer.

It should be added that the two passengers sitting in the backseat died at the place of the event. According to the available data, the cause of death of both individuals was severe multiorgan damage; moreover, the 17-year-old female sustained a spinal fracture, and the 28-year-old male had a fracture of the skull base.

## Materials and methods

### Biological materials

Sample of peripheral blood was collected from the driver and from two living passengers after accident. Autopsy blood was also taken from two dead victims during autopsy carried out within 24 h after death. The samples were kept frozen (−20 °C) until analyses. Blank blood samples for development and validation of analytical method were taken from unpoisoned subjects and from the Regional Centre of Blood Donation and Blood Treatment in Kraków.

### Standards and chemicals

Standard solutions of 1-(4-methoxyphenyl) piperazine (4-MeOPP), 1-(*m*-trifluoromethylphenyl) piperazine (TFMPP), 1-benzylpiperazine (BZP), 1-methyl-4-(phenylmethyl)-piperazine (MPMP), 2,5-dimethoxy-4-methylamphetamine (DOM), 2-methylmethcathinone (2-MMC), 3,4-dimethylmethcatinone (3,4-DMMC), 3,4-methylenedioxyamphetamine (MDA), 3,4-methylenedioxyethylamphetamine (MDEA), 3,4-methylenedioxymethyl-amphetamine (MDMA), 3,4-methylenedioxypyrovalerone (MDPV), 3,4-methylenedioxy-α-pyrrolidinobutiophenone (MDPBP), 3-methylmethcathinone (3-MMC), 4-bromo-2,5-dimethoxyamphetamine (DOB), 4-bromo-2,5-dimethoxyphenethylamine (2C-B), 4-chloromethcathinone, 4-fluoroamphetamine, 4-hydroxymethamphetamine, 4-methoxyamphetamine (PMA), 4-methoxymethamphetamine (PMMA), 4′-methoxy-α-pyrrolidinopropiophenone (MeOPPP), 4-methylethcathinone (4-MEC), 4-methylmethcathinone (4-MMC, mephedrone), 5-(2-aminopropyl) benzofuran (5-APB), 5-(2-ethylaminopropyl) benzofuran (5-EAPB), 5-(2-methylaminopropyl) benzofuran (5-MAPB), 6-(2-aminopropyl) benzofuran (6-APB), amphetamine, brephedrone, buphedrone, butylone, caffeine, cathinone, ephedrine, ephedrone, ethylphenidate, flephedrone, methamphetamine, methcathinone, methyl- benzylpiperazine, methylone, methylphenidate, *N,N*-dimethylamylamine, naphyrone, *N*-ethylbuphedrone (NEB), *N*-ethylcathinone, *N*-methyl-1,3-benzodioxolylbutanamine (MBDB), pentedrone, phentermine, phenylpropanolamine (norepherdrine), pseudoephedrine, α-PVP, and α-PVP-*d*_8_ used as internal standard (IS), were purchased from LGC Standards (Łomianki, Poland); ammonium formate, ammonium carbonate, formic acid, and acetic acid (all ≥99.9 % for LC-MS) from Sigma (Poznań, Poland); acetonitrile, methanol (all ≥99.9 % for LC–MS) and solid-phase extraction (SPE) columns LiChrolut RP-18 containing 500 mg C18-RP bonded silica from Merck (Warszawa, Poland).

### Extraction procedure

Each 1-mL sample was put into a clean 15-mL tube and diluted with 0.01 M carbonate buffer to pH 9.3 (1:5, v/v). α-PVP-*d*_8_ at a concentration of 0.1 mg/L was added to each sample. Then, the samples were vortexed and centrifuged for 7.5 min at 4400×*g*.

The SPE cartridge was firstly equilibrated with 1 mL each of methanol, distilled water, and the above carbonate buffer pH 9.3, and then the supernatant of each blood sample was applied on the cartridge and slowly passed through it. In the next step, matrix impurities were cleaned with 2 mL of the carbonate buffer pH 9.3, and then vacuum was applied to each cartridge for 30 min to remove residual moisture. The analytes were eluted with 2 mL of the 1 M acetic acid in methanol (1:9, v/v). The eluate was evaporated to dryness under a gentle nitrogen stream at 40 °C, and the residue was dissolved in 0.1 mL of a mixture of mobile phases (95 % phase A + 5 % phase B) and injected into a liquid chromatography–tandem mass spectrometry (LC–MS-MS) system.

The calibrators for quantitative analysis were prepared by spiking α-PVP **(**0.010, 0.025, 0.050, 0.250, and 0.500 mg/L) into the drug-free blood prior to extraction.

### Instrumentation

#### Liquid chromatography

An Agilent Technologies 1200 liquid chromatograph (Santa Clara, CA, USA) equipped with a binary pump (G1312 A) and an autosampler (G1329 A) was used. A 0.01 mL injection loop was used in the gradient mode. The chromatographic separation was performed with a Poroshell 120 EC-C18 column (100 × 3.0 mm id, particle size 2.7 µm, Agilent Technologies) and a Poroshell 120 EC-C18 guard column (5 × 3.0 mm id, Agilent Technologies). The phase A was water, which contained 0.2 % formic acid and 0.002 M of ammonium formate, and the phase B was acetonitrile with 0.2 % formic acid and 0.002 M of ammonium formate.

The gradient for samples concerning α-PVP and IS was programmed as follows: 95 %A/5 %B at a flow rate of 0.5 mL/min, followed by a linear change to 5 %A/95 %B at a flow rate of 1 mL/min in 10 min and held for 5 min.

#### Tandem mass spectrometry

A 6410 triple quadrupole mass spectrometer (Agilent Technologies) equipped with an electrospray ionization (ESI) source, operated under the positive mode was used. The operational parameters of the ESI source were as follows: vaporizing temperature, 350 ºC; pressure of the nebulising gas, 40 psi; flow of the drying gas, 9 L/min; capillary potential, 3.5 kV; fragmentor voltages, 84 V for α-PVP and 99 V for α-PVP-*d*_8_. The mass spectrometer was operated in the multiple reaction monitoring (MRM) mode with the transitions at *m*/*z* 232.1/91.1 (quantification) and 232.1/77.1 (qualification) for α-PVP and at *m*/*z* 240.3/91.1 and 240.3/77.1 for α-PVP-*d*_8_, respectively. The optimized collision-induced dissociation (CID) energies were 25 eV for transitions at *m*/*z* 232.1/91.1 (α-PVP) and at *m*/*z* 240.3/91.1 (α-PVP-*d*_8_), and 53 eV for transitions at *m*/*z* 232.1/77.1 (α-PVP) and at *m*/*z* 240.3/77.1 (α-PVP-*d*_8_).

### Validation of the LC–ESI-MS-MS method

#### Selectivity

To evaluate peak-purity and selectivity, blank blood samples (neither analyte nor IS added) were analyzed with each batch to check for impurity peaks that might interfere with detection of α-PVP or IS. To assess possible interferences of α-PVP, quality control (QC) samples were spiked individually to contain 10 mg/L each of 4-MeOPP, TFMPP, BZP, MPMP, DOM, 2-MMC, 3,4-DMMC, MDA, MDEA, MDMA, MDPV, MDPBP, 3-MMC, DOB, 2C-B, 4-chloromethcathinone, 4-fluoroamphetamine, 4-hydroxymethamphetamine, PMA, PMMA, MeOPPP, 4-MEC, 4-MMC, 5-APB, 5-EAPB, 5-MAPB, 6-APB, amphetamine, brephedrone, buphedrone, butylone, caffeine, cathinone, ephedrine, ephedrone, ethylphenidate, flephedrone, methamphetamine, methcathinone, methylbenzylpiperazine, methylone, methylphenidate, *N,N*-dimethylamylamine, naphyrone, NEB, *N*-ethylcathinone, MBDB, pentedrone, phentermine, phenylpropanolamine (norepherdrine), and pseudoephedrine.

#### Linearity and limits of quantification and detection

Calibration curves were constructed after the analysis of drug-free blood sample containing known amounts of α-PVP. To prepare these standards, blood samples were spiked with α-PVP at the following concentrations: 0.010, 0.025, 0.050, 0.250, and 0.500 mg/L. Each level was prepared in triplicate. The samples were extracted according to the procedure described before. Calibration curves were constructed by plotting the peak-area ratios (*y*) of the analyte (α-PVP)/(α-PVP-*d*_8_) against α-PVP concentration (*x*).

Validation samples were prepared in triplicate at the following concentrations: 0.020, 0.100, 0.500 mg/L of α-PVP to assess the accuracy of the method. Negative QC samples were analyzed after each linearity sample to evaluate potential carry-over. The limit of detection (LOD) of the method was determined by analyzing validation samples (*n* = 5) to determine if acceptance criteria were met for the analyte. The LOD was defined as the lowest concentration at which the analyte ion signal-to-noise ratio (determined by peak area) was ≥10. The limit of quantification (LOQ) was defined as the lowest concentration that met LOD criteria and also had a standard deviation within ±20 %.

#### Accuracy and precision

Inter- and intra-assay accuracy and precision data for α-PVP were determined with the low (0.020 mg/L), medium (0.100 mg/L), and high (0.500 mg/L) QC samples. Intra-assay data were assessed by comparing data from within one run (*n* = 5) and inter-assay data were determined with five separate runs (*n* = 15). Data were evaluated using one-way analysis of variance with day as the grouping variable. Accuracy, expressed as a percentage, was calculated by dividing the mean calculated concentration by the spiked concentrations, and multiplying by 100. Precision, expressed as percent relative standard deviation (%RSD), was determined by calculating the percent ratio of the standard deviation divided by the calculated mean concentration and multiplied by 100.

#### Matrix effect, extraction efficiency, and process efficiency

Extraction efficiency, matrix effect and process efficiency were evaluated via three sets of samples as described by Matuszewski [7]. Extraction efficiencies for α-PVP and IS were measured at each QC concentration. Blank blood was fortified with α-PVP and IS solution before and after SPE. Percent extraction efficiency from blood was expressed as mean analyte area of QC samples (*n* = 5) fortified with references standard solution before extraction divided by mean area of samples (*n* = 5) with references standard solution added after SPE. The matrix effect (relative) was calculated by comparing analyte peak areas obtained from six different blank blood samples fortified with α-PVP and IS solutions after SPE with peak areas of references standards solution at the same nominal concentrations prepared in a 95:5 (v/v) mixture of mobile phases A and B (neat samples). Matrix suppression or enhancement (absolute matrix effect) was calculated as follows: (mean peak area of fortified blood after SPE/mean peak area of the neat sample) × 100 − 100. The process efficiency shows the overall effect of SPE extraction efficiency and matrix effect on the quantification of analytes of interest. This was determined by comparing the mean analyte peak area of five samples fortified before SPE with the mean peak area of five neat samples prepared in the mobile phase.

#### Stability

Processed sample stability was investigated by reinjecting low, medium, and high QC extracted samples stored at 4 °C for 24, 48, and 72 h on the autosampler (*n* = 3) and at room temperature for 72 h and by calculating the results against the original calibration curve as a percent differences.

## Results

**Fig. 1:**
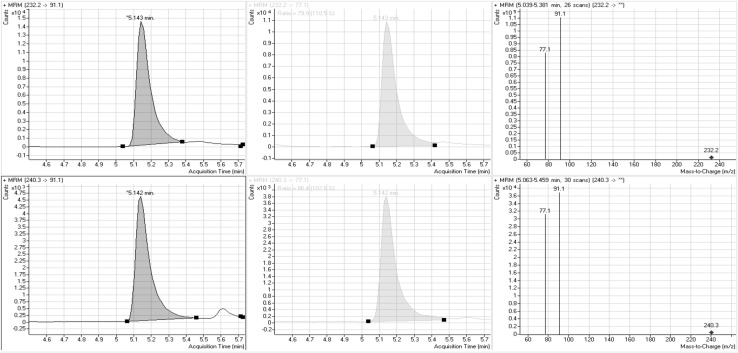
Multiple reaction monitoring chromatograms and product ion mass spectra of α-PVP (*top*) and α-PVP-*d*_8_ (*bottom*) extracted from an autopsy blood sample of passenger 3 recorded by liquid chromatography–electrospray ionization-tandem mass spectrometry

In the present study, we experienced five people who were involved in a traffic accident; among them, two were killed and three were injured, but survived the accident. It was disclosed that all of them had taken the NPS α-PVP. To accurately quantify α-PVP in their blood samples, we have established a detailed procedure for its analysis by LC–ESI-MS-MS together with the validation of the method. First of all, we tested the selectivity of this method using more than 50 of psychoactive compounds, but none of them interfered with the peck of α-PVP or IS (Fig. 1). The linearity of the calibration curve was confirmed in the range of 0.010–0.500 mg/L for α-PVP in blood samples; the regression equation and correlation coefficient *r* were : *y* = 0.0062*x* − 0.047, and *r* = 0.9994, respectively. The LOD and LOQ were 0.005 and 0.010 mg/L, respectively. Accuracy and precision data were generally satisfactory (Table 1).

**Table 1:** Accuracy and precision data of the developed method

Concentration of α-PVP (mg/L)	Intra-assay (*n* = 5)		Inter-assay (*n* = 15)	
	Accuracy (%)	Precision (%RSD)	Accuracy (%)	Precision (%RSD)
0.020	16.7	2.9	10.2	3.5
0.100	−0.8	3.0	−6.5	7.1
0.500	1.2	1.0	−0.9	1.8

*RSD* relative standard deviation

Because we used stable isotopic α-PVP as IS (isotope dilution method), it seems not necessary to present matrix effects and efficiencies. However, the data seem to be of interest for readers. The results showed that the method suffered from appreciable suppressive matrix effects (Table 2).

**Table 2:** Matrix effects, extraction efficiencies, and process efficiencies of the developed method

Compound	Blood concentration (mg/L)	Matrix effect (%)		Coefficient of variation (%RSD)	Extraction efficiency (%)	Process efficiency (%)
		Relative	Absolute suppression/enhancement			
α-PVP	0.020	0.24–0.36	−75.8 to −63.3	6.7	17.5–44.9	6.3–15.1
	0.100	0.25–0.35	−74.7 to −64.8	5.6	26.4–84.6	8.1–21.4
	0.500	0.31–0.35	−68.8 to −64.8	2.2	57.4–77.1	19.4–25.9
α-PVP-*d*_8_	0.100	0.33–0.45	−67.5 to −54.7	7.7	31.0–87.0	11.8–28.3

The stability of α-PVP at 4 °C and room temperature showed that this compound is relatively stable (Table 3).

**Table 3:** Stability of α-PVP

Compound	Blood concentration (mg/L)	Percent difference			
		4 °C			Room temperature
		24 h	48 h	72 h	72 h
α-PVP	0.020	−0.16	−7.0	11.9	6.8
	0.100	−2.1	−11.5	11.6	11.5
	0.500	−0.48	−2.6	14.0	12.5
α-PVP-*d*_8_	0.100	−3.9	−8.3	11.5	5.7

In blood samples collected from the surviving and deceased participants in the accident, the analysis demonstrated α-PVP in a diversified range of concentration values. The results of toxicological tests performed in individuals involved in the accident are presented in Table 4.

**Table 4:** Toxicology results of the participants in the accident

Individual	Blood collection time	Concentration of α-PVP (ng/mL)	Time	Post-accident alcohol blood level (‰)
Living subjects				
Driver, male—21 years	9:30 a.m. 10:30 a.m.	230 360	6:45 a.m.	0.0
Passenger 1, male—36 years, front seat	10:10 a.m. 01:30 p.m.	30 20	6:50 a.m.	1.2
Passenger 2, female—19 years, back seat, center	–^a^	–^a^	7:10 a.m.	0.4 (medical records)
Autopsy subjects				
Passenger 3, female—17 years back seat, side	–	650	–	1.9
Passenger 4, male—28 years back seat, side	–	290	–	2.6

^a^Not sampled

The blood samples were collected from the driver 3.5 and 4.5 h after the accident, arriving at an increasing sequence of α-PVP concentration values of 230 and 360 ng/mL, respectively. As it follows from data originating from the court records, the male insufflated the psychoactive twice in the period preceding the accident, i.e., ~6 and 2 h previously, which explains the results as the effect of interposition of the elimination and absorption phases of both doses taken at different times. The demonstrated concentrations of α-PVP in the blood of the driver should be considered high as compared with the literature data available [4, 8]. It may be also assumed that at the time of the accident, the blood α-PVP level of the driver might have been higher than the level demonstrated in the tests. Passenger 1, a survivor of the accident, had taken α-PVP ~7.5 and 13.5 h before blood collection, and after that period, the substance continued to be present in his blood at 30 and 20 ng/mL, respectively. On the other hand, in the blood of the deceased individuals involved in the accident, α-PVP concentration values were high (650 and 290 ng/mL), because they reflected the levels of the drug after 6 and 2 h after insufflating α-PVP doses. The numerical values were different, which most likely resulted from the difference in the size of doses apportioned to both abusers using the “hit-and-miss” method and in their body parameters.

## Discussion

In the analyzed case, chemical and toxicological tests demonstrated a psychoactive substance α-PVP in the blood samples collected from the driver and perpetrators of the accident. The alleged substance belongs to the group of NPSs, being in the category of synthetic cathinone derivatives. Until 2015, α-PVP was not included in the list of narcotic substances and psychotropic agents of the legal regulations in force [9]. The cathinone α-PVP belongs to the group of pyrrolidinophenones. The group also includes two structural α-PVP analogues, namely pyrovalerone and MDPV, which are included in the list of psychotropic substances in the above regulation.

Although α-PVP was synthesized in the 1960s, at the present time it has gained some popularity in the narcotic market. Among various types of cathinones, derivatives of α-PVP have become popular as NPSs due to the presence of the lipophilic pyrrolidinyl group that increases the ability to cross the blood-brain barrier to obtain a better psychedelic effect [10, 11].

The mechanism underlying α-PVP activities is poorly understood, and for this reason, while commenting on the biological activities of the compound, the likeness of its activities to those of other compounds is also taken into consideration, namely pyrovalerone and especially MDPV, which show close similarity in their chemical structures and allow assuming that they may be similar to their biological activities. Generally, all three above-mentioned compounds belong to selective reuptake inhibitors of neurotransmitters dopamine, noradrenaline, and serotonin. In view of their mechanism of activity, pyrrolidinophenones are considered central nervous system stimulants. As a rule, the basic anticipated symptoms of people often taking the above compounds include enhanced energy, euphoria, empathy, openness, and increased libido. Nevertheless, soon afterwards, there may appear such negative side effects as tachycardia, increased arterial pressure, confusion, agitation, insomnia, aggression, and hallucinations [4].

The employed α-PVP doses have not been precisely determined. However, based on the presently analyzed case and data derived from Internet forums frequented by young people, there may be a fairly broad range of 100–500 mg.

The available literature described that as compared to cocaine and amphetamine, which belong to the group of classic central nervous system stimulants, both α-PVP and its structurally close derivative MDPV are much more potent selective reuptake inhibitors of dopamine and noradrenaline. Moreover, in studies performed in mice, a significant increase in locomotor activity by the compounds was demonstrated, being higher than that by cocaine and amphetamine and resulting from an increased dopamine level [12]. Psychophysical tests employed in assessment of ability to operate motor vehicles and performed in individuals under the influence of MDPV demonstrated considerable functional impairment of psychomotor abilities in 84 % of testees, and in 7 %, there was moderate or complete inability to operate motor vehicles. The determined blood MDPV concentration values in the tested individuals were below 500 ng/mL [12]. In view of a lack of data on toxic α-PVP levels, interpretation of toxicology results in the analyzed case is most assuredly complex and ambiguous.

Knoy et al. [4] described a case of a driver under the influence of α-PVP. The driver was stopped by the police due to bravado driving; following the intervention, the policemen noticed symptoms that might suggest his being under the influence of a narcotic substance, namely verbal confusion, disorientation, excitation, tachycardia, dilated pupils, and tremors. The symptoms observed in the course of the above police intervention are similar to those previously noted in cases of other representatives of the pyrrolidinophenone group. During subsequent procedures involving the above driver, his blood sample collected ~2 h after the police intervention demonstrated α-PVP at a concentration of 63 ng/mL, being thus several times lower than the values noted in the presently analyzed driver case (α-PVP 230 and 360 ng/mL).

Analyzing possible behavior patterns of individuals under the influence of α-PVP, one may also quote other cases that well illustrate the patterns, although they are not related to traffic. Nagai et al. [8] provide a description of a male who exhibited sudden violent behavior at a place where several individuals lived together. The roommates tried to restrain the excited man, who collapsed after approximately 2 h of struggling, and after 30 min of routine resuscitation efforts, he died. His restraint was thought to induce death from asphyxia due to chest compression. During autopsy, intra-neck muscle hemorrhage, submental and high neck petechiae, and facial congestion were observed. Toxicological analysis of a postmortem blood sample demonstrated 411 ng/mL of α-PVP.

The present authors experienced a case of a death of a 19-year-old man with an underlying mechanism similar to that described above [13]. Because of his aggressive and dangerous behavior, the man was brought to a mental hospital by the police. In the hospital, following a short period of calming down, he developed progressive motor agitation and started to destroy a patient room, and it was necessary to restrain him using immobilization belts. In spite of intense efforts at resuscitation, the patient died of acute circulatory and respiratory failure associated with psychomotor excitation. A postmortem blood sample showed α-PVP at the level of 850 ng/mL.

In both above cases, the mechanism of death should be considered within the category of complications of pathological motor excitation. The mechanism is most commonly encountered in individuals who are incapable of controlling their behavior, being under the influence of psychoactive substances or exhibiting alcohol delirium. Pathological excitation causes the affected individual to thrash around purposelessly, chaotically employing all muscles at the same time, which markedly intensifies oxygen consumption.

Nagai et al. [8] drew attention to the danger of employing sustained restraint of excited persons with hallucinations resulting from α-PVP or other stimulants, suggesting the use of sedation and adrenergic blockade. Thus, as it follows from the practice of the present authors [13] and published literature data [8], as compared to methamphetamine and MDMA, which can be lethal, α-PVP and related drugs appear to be less lethal, but seem more hallucinogenic and agitating.

The metabolism of α-PVP may be fairly slow. Such a thesis results from observations of its concentration values demonstrated for example in the blood of the participants in the traffic accident. As shown in Table 4, in the surviving passenger 1, the compound (20 ng/mL) was detected, even 13.5 h after his having taken the last dose.

Analyzing the available court records pertaining to the present case, including the driver’s medical and psychological examination results, there is no doubt that at the time of the accident, he might have lost normal psychomotor ability resulting in increased energy and euphoria, and might have been agitated and unable to control his car fully; he also might have had a brief episode of unconsciousness. Moreover, the behaviors of the passengers who were also under the influence of the drug might have influenced the decreased alertness of the driver while driving a car. The very well-documented description of the behavior of the driver accused of causing a traffic accident, being under the influence of α-PVP and being based mainly on the explanations that he provided is in accordance with the classic features of the substance effects observed in other individuals and described in the literature. Additionally, attention was focused on the mental aspect of the analyzed case, which cannot be unrelated to the tragic accident.

## Conclusions

The presented case report points to a unique opportunity to understand the psychological effects of α-PVP in a real driving situation, emphasizing—along with another report [4]—the importance of police observation on the road, especially when alcohol and/or classic narcotic substances have been ruled out at the site.

## References

[1] DRUID (2012) 6th Framework programme. Final report: work performer, main results and recommendations. http://www.druid-project.eu. Accessed August 2012

[2] Heis T, Lyckegaard A, Simonsen KW, Steentoft A, Bernhoft IM (2013). Risk of severe driver injury by driving with psychoactive substances. Accid Anal Prev.

[3] Musshoff F, Madea B, Kernbach-Wighton G, Bicker W, Kneisel S, Hutter M, Auwärter V (2014). Driving under influence of synthetic cannabinoids (“Spice”): a case series. Int J Legal Med.

[4] Knoy JL, Peterson BL, Couper FJ (2014). Suspected impaired driving case involving α-pyrrolidinovalerophenone, methylone and ethylone. J Anal Toxicol.

[5] Maas A, Wippich C, Madea B, Hess C (2015). Driving under the influence of synthetic phenethylamines: a case series. Int J Legal Med.

[6] Karinen R, Tuv SS, Oiestad EL, Vindenes V (2015). Concentrations of APINACA, 5F-APINACA, UR-144 and its degradant product in blood samples from six impaired drivers compared to previous reported concentrations of other synthetic cannabinoids. Forensic Sci Int.

[7] Matuszewski BK (2006). Standard line slopes as a measure of relative matrix effect in quantitative HPLC-MS bioanalysis. J Chromatogr B.

[8] Nagai H, Saka K, Nakajima M, Maeda H, Kuroda R, Igarashi A, Tsujimura-Ito T, Nara A, Komori M, Yoshida K (2014). Sudden death after sustained restraint following self-administration of the designer drug α-pyrrolidinovalerophenone. Int J Cardiol.

[9] The law on preventing drug addiction dated 29 July 2005 (2012) Ustawa o przeciwdziałaniu narkomanii z dnia 29 lipca 2005 r. (tekst jednolity: Dz. U. z 2012 r. poz. 124 z poź. zm.)

[10] Zaitsu K, Katagi M, Tatsuno M, Tsuchihashi H, Ishii A (2014). Recently abused synthetic cathinones, α-pyrrolidinophenone derivatives: a review of their pharmacology, acute toxicity and metabolism. Forensic Toxicol.

[11] Wurita A, Hasegawa K, Minakata K, Gonmori K, Nozawa H, Yamagishi I, Susuki O, Watanabe K (2014). Postmortem distribution of α-pyrrolidinobutiophenone in body fluids and solid tissues of a human cadaver. Leg Med.

[12] Kaizaki A, Tanaka S, Numazawa S (2014). New recreational drug 1-phenyl-2-(1-pyrrolidinyl)-1-pentanone (alpha-PVP) activates central nervous system via dopaminergic neuron. J Toxicol Sci.

[13] Konopka T (2014) Archives of Department of Forensic Medicine Jagiellonian University Medical College in Krakow (S-626)

